# Ecological lipidology

**DOI:** 10.7554/eLife.79288

**Published:** 2022-09-07

**Authors:** Laura Christin Trautenberg, Marko Brankatschk, Andrej Shevchenko, Stuart Wigby, Klaus Reinhardt

**Affiliations:** 1 Biotechnology Center (BIOTEC), Technische Universität Dresden Dresden Germany; 2 Max Planck Institute of Molecular Cell Biology and Genetics Dresden Germany; 3 Applied Zoology, Technische Universität Dresden Dresden Germany; 4 Department of Evolution, Ecology and Behaviour, Institute of Infection, Veterinary and Ecological Sciences, University of Liverpool Liverpool United Kingdom; Pennsylvania State University United States; Pennsylvania State University United States

**Keywords:** diet choice, fatty acids, fitness, food web, membrane, sterols

## Abstract

Dietary lipids (DLs), particularly sterols and fatty acids, are precursors for endogenous lipids that, unusually for macronutrients, shape cellular and organismal function long after ingestion. These functions – cell membrane structure, intracellular signalling, and hormonal activity – vary with the identity of DLs, and scale up to influence health, survival, and reproductive fitness, thereby affecting evolutionary change. Our Ecological Lipidology approach integrates biochemical mechanisms and molecular cell biology into evolution and nutritional ecology. It exposes our need to understand environmental impacts on lipidomes, the lipid specificity of cell functions, and predicts the evolution of lipid-based diet choices. Broad interdisciplinary implications of Ecological Lipidology include food web alterations, species responses to environmental change, as well as sex differences and lifestyle impacts on human nutrition, and opportunities for DL-based therapies.

## Introduction

The importance for health and disease of optimal levels of total and individual dietary lipids (DLs) is uncontested in medical research: links to obesity, diabetes, cardiovascular and neurodegenerative disease, and cancer have been much studied and commented upon (Ludwig et al., 2018). For example, the over-consumption of dietary saturated fatty acids (SFAs) is thought to contribute to obesity-related pathology, while phytosterol consumption is thought to be beneficial, reducing harmfully high cholesterol levels by blocking uptake (Wang and Hu, 2017). However, beyond excess consumption in humans, or improved feed production for livestock and fisheries, the effect of DLs has major ecological and evolutionary implications in research areas ranging from food web structure, species distribution, to sex differences and diet choice.

**Figure 1.**
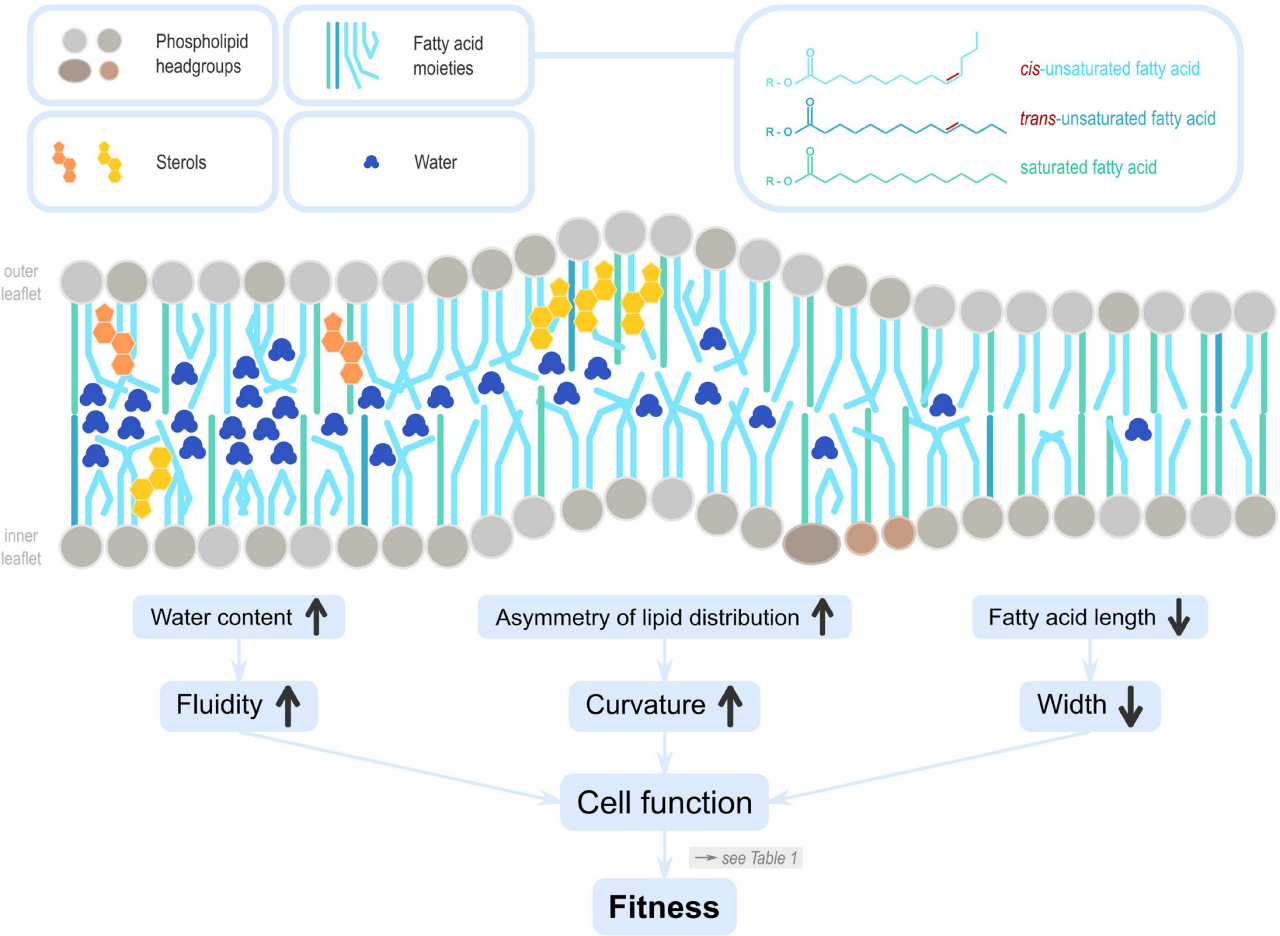
Lipids influence cell membrane properties. Dietary lipids (DLs) integrate into cell membranes where they have vital cellular functions that ultimately influence reproductive fitness (see Table 1). Phosphoglycerolipids are polar, with a hydrophilic ‘head’ and hydrophobic ‘tail’. In cell membranes, they are oriented with heads on the outside to the extracellular environment, the tails on the inside towards the cell interior. Sterols are also incorporated, and can be locally enriched to affect membrane properties in specific areas. The lipid profile differs between the inner and outer leaflets, reflecting their different biological functions. The lipid profile of a membrane determines its diffusion properties, fluidity, curvature, and width (Harayama and Riezman, 2018). The quantity of membrane sterols, as well as the amount, chain length, and saturation, of fatty acids that form phospholipids and sphingolipids influence the amount of water interspersed in the membrane leaflets. For example, cis unsaturation in the tails of fatty acids change the angle between neighbouring carbons and generate a kink, creating space for water molecules to intersperse, which in turn increases membrane fluidity. Similarly, the unequal distribution of sterols in the leaflets can result in asymmetries (Steck and Lange, 2018), and facilitate membrane curvature. Curvature determines molecular transport and endocytosis but reduces stiffness and integrity, and its maintenance likely incurs energetic costs (Stachowiak et al., 2013). Membrane width is mostly defined by fatty acid chain length, however, their saturation type and abundance is also important. Membrane width influences the insertion of transmembrane proteins and is thus a critical variable for the protein sorting machinery in the cellular organelles, including the Golgi apparatus or the endoplasmatic reticulum.

In nutritional ecology, the energetic value of DLs is well established (Raubenheimer et al., 2009; Simpson and Raubenheimer, 2012). Beyond that, DLs critically influence other bodily functions such as membrane properties (Figure 1; Behmer and Nes, 2003; Hazel, 1995; Hulbert et al., 2014; Jie et al., 2019; Kostal, 2010; Sinensky, 1974; Twining et al., 2021), intracellular signalling, and hormonal activity (Lavrynenko et al., 2015; Yore et al., 2014). One general and key feature makes DLs unusual among nutrients: whilst consumers break down some DLs into basic molecules (e.g., free fatty acids [FAs]) but not others (sterols), the incorporation of these basic molecules into their own lipids (‘building blocks’) shapes biological functions long after ingestion, from hours (muscle) to years (brain). DLs, and the ability to process them from other dietary material, vary through consumer hierarchies and can vary across environments. Many organisms cannot synthesise all lipids themselves, that is, they are auxotroph for certain lipid classes (e.g., sterols) or lipid species (e.g., polyunsaturated fatty acids [PUFAs] or glycerophospholipids with PUFA moieties) that are required for organismal function (essential lipids) (Twining et al., 2021) . For example, insects and nematodes are sterol auxotrophs (Behmer and Nes, 2003); many vertebrates, including humans, but also insects are restricted in their PUFA production, and some can produce monounsaturated fatty acids (MUFAs) only. Understanding this paradox and how DL-mediated molecular and cell functions affect reproductive fitness (the number and quality of offspring produced in the next generation) in different species, that then have consequences for evolution and food webs, requires a joined-up approach of lipid cell biology, nutrition, and ecology, which we term Ecological Lipidology.

The Ecological Lipidology approach can be viewed as part of the broader nutritional ecology framework (Raubenheimer et al., 2009; Simpson and Raubenheimer, 2012). It augments this and previous nutritional approaches (reviewed in Raubenheimer et al., 2009) by being very explicit in terms of lipid species or lipid classes and their molecular or cell biological mechanism (the lipidology part). Ecological Lipidology also links specific lipids and their functions to the phenotype and fitness, by examining how the environment causes changes in the lipidome and by analysing ecological and evolutionary consequences of such lipidome variation (the ecological part). Because DLs are not essential for energy metabolism, the energetic aspect is currently underrepresented in our Ecological Lipidology approach.

The advent of lipidomics, the organism-wide quantitative analysis of the full lipid complement (Ejsing et al., 2009), has led to an unprecedented availability of data on the identity and abundances of individual species from major lipid classes. In this review we emphasise that distinct DLs have essential non-metabolic functions that are important from the cellular to the ecological level. We present the Ecological Lipidology approach by (1) summarising that different environments cause variation in the lipidome of producers. We (2) review the strong cellular, organismal, survival, and fitness impact of DL variation and show (3) that the impacts can depend on the environment. This leads us to (4) testing the prediction that natural selection favours lipid selectivity by diet choice, which itself may be regulated by the environment. We (5) propose lipid selectivity to have wide-ranging consequences for food web ecology, biodiversity, micro- and macroevolution. We conclude by proposing some implications of Ecological Lipidology for biomedical research.

## Environmental influences on lipidome properties of producers

Primary producers in food webs such as bacteria, protists, algae, fungi, and plants use carbohydrates from the environment to make a diverse range of lipids, such as FA, sterols, or sterol-like molecules. These organisms show large interspecific (Surma et al., 2021) variation in levels of both gross lipid classes and individual lipid species (e.g., de Carvalho and Caramujo, 2018; Kainz et al., 2009; Twining et al., 2021; Zu et al., 2021). Insufficient data exist but it appears that producers rarely alter their lipidome in response to pollinators (Zu et al., 2021), parasites (Eigenbrode and Espelie, 1995), or predators, that is, in response to ecological top-down processes. Instead, different nutrient availability, specialised evolved metabolic pathways, or adjustment of the production of specific lipid species or lipid classes in response to the environment, particularly temperature (Wang et al., 2006), shape lipidomes. A prominent, 50-year-old theory, named ‘homeoviscous adaptation’, posits that ectothermic organisms modify the biophysical properties of cell membranes (Figure 1) by incorporating specific lipids that then increase survival under cold temperatures (Brankatschk et al., 2018; Geiser, 2021; Hazel, 1995; Hulbert et al., 2014). These modifications refer to within-individual, reversible changes that typically happen within hours, rather than to genetic changes over generations that evolutionary biology defines as ‘adaptation’. We therefore refer to the theory as ‘homeoviscous plasticity’. Sterols and FAs – incorporated, for example into glycerophospholipids or sphingolipids – determine major properties of cell membranes (Figure 1), and are key players in homeoviscous plasticity in two ways. First, at low temperatures lipids organise themselves into local lipid microdomains, some of which can recruit membrane proteins and may render them inactive (Lin et al., 2018). Second, producers can counter the decreased membrane fluidity and permeability at low temperatures (Brankatschk et al., 2018; Caron et al., 2014) by incorporating glycerophospholipids that consist of long and unsaturated FA moieties or by incorporating sterols. This leads to increased membrane width (Figure 1; Caron et al., 2014). Some lipids are better than others at maintaining cellular water balance under cold temperatures because the physical shape of membrane lipids affects how they are organised, and the ability of water to intersperse (Garda et al., 1997; Rogowska and Szakiel, 1988; Figure 1). However, in deep freeze conditions, higher water content may increase cellular damage due to ice-crystal formation (Giraud et al., 2000; Turk et al., 2011). This and disadvantages of cold resistance at higher temperatures (Brankatschk et al., 2018; Hulbert and Abbott, 2011; Twining et al., 2021; Zeis et al., 2019) likely prevent the evolution of genetically fixed responses in lipid composition to cold temperatures and maintain homeoviscous plasticity.

In summary, the lipid composition of membranes has profound consequences for membrane properties and function (Coskun and Simons, 2012). Producers modulate their lipidome to match membrane function to temperature challenges (homeoviscous plasticity); the advantages in other environments, such as heat, drought, circadian changes, or intracellular parasites, are currently less clear (Scheitz et al., 2013; Tognini et al., 2017; Twining et al., 2021).

## Impact on consumers: individual DLs shape cellular traits that scale up to fitness

The membranes of producers and consumers may have little in common chemically but they still have to function as barriers. Consumers modify their endogenous lipids for the same reasons as producers: to build cell membranes that maintain fluidity in the face of environmental variation (Hulbert et al., 2014; Hulbert and Abbott, 2011). Given the large variation in the lipidome of producers and in the consumers’ ability to convert DLs into complex lipids (Behmer and Nes, 2003; Clayton, 1964; Jing and Behmer, 2020; Twining et al., 2021), we expect that specific DLs exert large differences on consumers. This expectation has strong support from cellular to fitness levels and includes differences caused by the absence or the amount of a specific DL (quantitative effects) but also qualitative effects, when one DL species is replaced by another.

### Quantitative and absence effects of DL

Hundreds of studies demonstrate that absence or suboptimal amounts of certain DLs affect cell function, physiology, or health. Most focus on calorically undefined high-fat and low-fat diets or n-3/n-6 PUFA ratios, especially in metabolism and obesity of mammals (Moussavi et al., 2008) but ecological effects are also demonstrated for different species and other lipid classes. The concentration of dietary cholesterol and other sterols alters growth and fecundity in many arthropod species (Ilić et al., 2019; Jing and Behmer, 2020). Stearic acid provided with the diet and incorporated into endogenous lipids activates the JNK pathway, markedly improves mitochondrial function and biogenesis in human cells and *Drosophila melanogaster*, translating into longevity benefits and the rescue of genetic defects (Bajracharya and Ballard, 2018; Senyilmaz Tiebe et al., 2018; Senyilmaz et al., 2015). PUFA reduce coral bleaching (Tagliafico et al., 2007), improve immune defence, survival, growth, and development of birds (Awadin et al., 2020; Twining et al., 2016), or critically influencing many aspects of female reproduction (Schultzhaus et al., 2018; Schultzhaus and Carney, 2017; Wathes et al., 2017). PUFA supplementation alters the sperm lipidome and sperm quality parameters in many species but evidence for impacts on fertility or fitness remains scarce (rats: Yan et al., 2013, guppies: Rahman et al., 2014). These examples illustrate that specific levels of certain lipids are required and that natural selection can be expected to operate on maintaining the ingestion of appropriate quantities of DLs. An important issue in Ecological Lipidology is to determine how essential a DL’s effect is on fitness, or whether it can be replaced by alternatives. The examples below show that replacing lipids as they become scarce is not always an option because lipid-mediated reductions in bodily functions can impact fitness.

### Qualitative variation

#### Cellular effects, metabolism, growth, and development

The chemical properties of DLs incorporated as endogenous lipids into membranes (Figure 1) can fundamentally change the function of even differentiated cells (Stinkens et al., 2015; Table 1). This is particularly relevant in gut cells because they are the first contact points, they differ in their absorption rates of different sterol species in humans, and because DL effects can change subsequent lipid uptake. In *D. melanogaster*, dietary sterols increase the number of endocrine cells in the midgut, whereas stearic acid decreases, or even kills them (Obniski et al., 2018), impairing future food uptake and water homeostasis. In mice, metabolised dietary PUFAs are preferentially built into the complex phospholipids of membranes as soon as they are available (Levental et al., 2020) and PUFA-enriched phosphatidylcholine of the apical (luminal) membrane of gut cells increases the absorptive function of enterocytes, compared to SFA (Wang et al., 2016). Other cellular changes with likely fitness effects are summarised in Table 1.

**Table 1.** Examples of specific dietary lipids affecting animal health and fitness traits. Potential fitness effects for cellular and metabolic traits as well as the given or putative lipid activity (signalling **S**, membrane property **M**, or unknown **?** changes) are given in brackets. Rat – *Rattus rattus*, mouse – *Mus musculus*, fly – *Drosophila melanogaster*, worm – *Caenorhabditis elegans*. * indicates that studies provide evidence for the precise molecular mechanism. See bottom of table for abbreviations of lipids.

DL treatment (vs. control)	EFFECT of TREATMENT	SPECIES	SOURCE
**CELLULAR TRAITS and METABOLISM**			
**DHA C22:6** (vs. cholesterol)	Modulates enterocyte miRNAi 107 expression (alteration of circadian rhythm), (**S**)	Human Caco2 cell culture	Daimiel-Ruiz et al., 2015
**5-PAHSA** (vs. 9-PAHSA)	Stimulates insulin secretion in the pancreas, facilitates glucose transport, anti-inflammatory (higher metabolic rate), (**S**)	Mouse	Yore et al., 2014*
**DHA C22:6/EPA C20:5** (vs. other FAs incl. C14:0, C16:0)	Stimulated insulin secretion, facilitates glucose transport, anti-inflammatory (higher metabolic rates), (**S**)	Mouse	Oh et al., 2013*
**DHA C22:6** (vs. LA C18:2)	Reduces mitochondrial activity (reduced metabolic rate, reduced oxidative stress), (**S**)	Mammalian cells	Sullivan et al., 2018*
**Lard** (vs. fish oil)	Increases ROS production, insulin resistance, mitochondrial dysfunction, oxidative stress, and mitochondrial fission (increased lifespan or reproduction), (**S**)	Mouse, rat	Chen et al., 2012; Lionetti et al., 2014; Yu et al., 2014
**PA C16:0, POA C16:1** (vs. OA C18:1, LA C18:2)	Reduces growth of dividing cells (slow development), (**S**)	Mouse, cell lines	Lien et al., 2021
**DEFENCE, GROWTH, and DEVELOPMENT**			
**Long-chained SFA** (vs. SFA)	Reduces growth hormone production (**S**)	Mouse	Levi et al., 2015*
**Fish oil** (vs. lard)	Reduces enteric damage during infections (**M**)	Mouse	DeCoffe et al., 2016
**Sitosterol, stigmasterol, campesterol** (vs. cholesterol)	Reduces body size and weight (**S**)	Fly	Lavrynenko et al., 2015*
**Brassicasterol, cholestanol, zymosterol, desmosterol** (vs. sitosterol, stigmasterol, campesterol, cholesterol)	Prevents larval or pupal development because they were no precursors for ecdysteroid hormones (**S**)	Fly	Lavrynenko et al., 2015
**POA C16:1** (vs. OA C18:1, PA C16:0; AA C20:4; EPA C20:5; DHA C22:6)	Modulates IGF1 signalling that controls growth and proliferation of white adipose tissue (**S**)	Mouse	Meln et al., 2019
**Plant PUFA** (vs. yeast PUFA)	Increases developmental rates at 12°C, reduces rates at high temperatures (**S**, **M**)	Fly	Brankatschk et al., 2018
**PHYSIOLOGY, BEHAVIOUR, and HEALTH**			
**MUFA** (vs. SFA) **n-3** PUFA (vs. n-6 PUFA)	Reduces obesity (**S**, **M**)	Human	Moussavi et al., 2008
**EPA 20:5, DPA 22:5, DHA C22:6** (vs. ALA C18:3)	Increases levels of long-chain n-3 PUFA (C20-22) in the blood thereby delaying mortality (**S**)	Human	Harris et al., 2021
**SFAs** (vs. MUFAs)	Modulates dopaminergic signalling thereby increasing locomotory activity (**S**)	Rat	Hryhorczuk et al., 2016
**AA C20:4 and DGLA C20:3** (vs. EPA C20:5)	Promotes resistance to starvation and extends lifespan by increased autophagy (**S**)	Worm	O’Rourke et al., 2013
**Enriched ALA C18:3** (vs. enriched LA C18:2)	Prevents hibernation (**?**)	Marmot *Marmota flaviventris*	Hill and Florant, 2000
**PUFA** (vs. SFA)	Individuals select colder areas that reduce body temperature (**?**)	Several species of lizard	Simandle et al., 2001
**REPRODUCTION, FERTILITY, and FITNESS**			
**Stigmasterol** (vs. cholesterol, campesterol, or sitosterol)	Reduces male fertility (**S**)	Ladybird beetle *Coccinella septempunctata*	Ugine et al., 2022b
**Fish oil** (vs. corn oil)	Increases fertilising ability of sperm (**M**)	Chicken *Gallus domesticus*	Blesbois et al., 1997
**Plant-based lipids** (vs. yeast-based lipids)	Delays sperm production, decreases sperm viability, reduces sperm ROS production rate, no effect on sperm osmotic stress resistance (**S, M**)	Fly	Guo and Reinhardt, 2020
**DGLA C20:3** (vs. OA 18:1)	Causes sterility via germ-cell ferroptosis (**S**)	Worm, human	Perez et al., 2013
**EPOA C20:5** (vs. ARA C20:4)	No difference in clutch sizes (**S**)	*Daphnia magna* and *Daphnia pulex*	Ilić et al., 2019
**ALA C18:3** (vs. PA C16:0)	Reduces reproductive rate, offspring size, and survival (**S**)	Hydra, *Hydra oligactis*	Kaliszewicz et al., 2018
**OA C18:1** (vs. LA C18:2, VCA C18:1, DGLA C20:3, EPA C20:5)	Rescues mating-induced reduction in female lifespan (**?**)	Worm	Choi et al., 2021

AA – arachidonic acid, ALA – α-Linolenic acid, DGLA – bihomo-γ-linolenic acid, DHA – docosahexaenoic acid, DPA – docosapentaenoic acid, EPA – eicosapentaenoic acid, LA – linoleic acid, OA – oleic acid, PA – palmitic acid, PAHSA – palmitic acid esters of hydroxystearic acid, POA – palmitoleic acid, VCA – *cis*-vaccenic acid.

Specific DLs fundamentally affect growth and development of arthropods (Table 1, Jing and Behmer, 2020), of other ectotherms, and of fermenting yeast (Liu et al., 2019). These effects on growth are mediated mainly by structural cell membrane alterations and hormonal changes (Lavrynenko et al., 2015). As dietary FAs associate with key morphogens (molecules that control patterning during development) such as Hedgehog and Wnt, a strong prediction – but a key unknown – is that developmental variation arises because DLs alter the structure of morphogens.

DL-derived lipids act as ligands in metabolic signalling cascades and alter metabolic rates (Yang et al., 2018). This fact may explain why somatic growth increases when DLs lower mass-specific resting metabolic rates (Ruiz et al., 1988). Other metabolic differences may arise because DLs mediate membrane fluidity around Na+ pumps that directly affect ATP production (Hulbert and Abbott, 2011).

Finally, DLs can enter the lipidome of the mitochondrial membrane, altering mitochondrial structure and function. Thereby, DLs change metabolic rates in some mammals and birds (Geiser, 1990; Hulbert and Abbott, 2011; Twining et al., 2016; Vasam et al., 2019) but not in others (Fuller and Rendon, 1977; Table 1). The DL-mediated metabolic effects on growth are more pronounced in ectothermic than endothermic vertebrates (e.g., Stickney and Andrews, 1972; Tidwell et al., 2007) but few studies consider whether these metabolic changes are adaptive, such as under different temperatures, or caloric restriction.

#### Immunity, physiology, health, and behaviour

DLs cause immune defence differences at the cellular and physiological level (López-Fandiño, 2020; Twining et al., 2016; Weill et al., 2020; Table 1) but also at the ecological level of host recognition. Many parasitic and pathogenic viruses, bacteria, and eukaryotes require specific surface lipids of the host to recognise, enter, or reproduce on the host. DLs that are directly incorporated into endogenous lipids occur on the skin and may, therefore, mediate the host cues that parasites require (Eigenbrode and Espelie, 1995).

In addition to cold resistance, DLs cause wide-ranging physiological and health effects (Table 1; Hulbert et al., 2014; Ludwig et al., 2018; Twining et al., 2021). Replacing n-3 with n-6 PUFA beneficially extends hibernation bouts and lowers body temperatures of hibernating mammals (Hulbert and Abbott, 2011; Table 1). DL effects on aging and lifespan are complex (Hulbert, 2005) but mammalian and avian lifespans often appear insensitive to DLs (Hulbert and Abbott, 2011; Naudí et al., 2013). Most aging data are correlative, compare non-isocaloric diets, or do not correct for different environments (e.g., in humans, Mediterranean food in southern Europa compared to Western-type food in central Europa). However, controlled lab experiments with female *D. melanogaster* revealed that limiting quantities of sterols (cholesterol) regulate the well-known effects of carbohydrates and protein on lifespan (Zanco et al., 2021), suggesting that lipids may often interact with other nutrients in their effects on lifespan. Finally, many DLs influence symbionts with downstream effects on health or performance (Clayton, 1964; Schoeler and Caesar, 2019).

#### Reproduction, fertility, and fitness

Different dietary sterols are incorporated into reproductive tissues with different speed and extent in *D. melanogaster* (Knittelfelder et al., 2020). In ladybird beetles, where males deprived of dietary sterols become completely infertile (Ugine et al., 2022b), individual dietary sterols rescue fertility with different efficacies (Table 1). In vertebrates, some sperm functional deficiencies based on dietary cholesterol can be rescued by other dietary sterols (Saez and Drevet, 2019), suggesting low sterol specificity. Female *D. melanogaster* fed with increased yeast sterols had larger ovaries than plant-sterol-fed ones (Knittelfelder et al., 2020). No data seem to exist on the efficacy of sex hormones built from different sterol precursors.

For most cases the molecular mechanisms underlying the observed effects are unknown (Table 1). However, taken together, there is widespread evidence that specific DLs limit or mediate key body functions, health, and reproductive fitness (Table 1).

## Environment-dependent lipid effects on fitness and lipid choice

DL effects on fitness-related traits predict that natural selection favours mechanisms that (1) protect from energy metabolism use those lipids that have structurally important functions, and that (2) facilitate lipid-selective foraging. Moreover, because lipid function is environment-dependent (e.g., temperature), lipid choice is predicted to be also environment-dependent. This topic has received less scrutiny (Table 1) but is likely important: given that lipid properties in consumer membranes provide similar benefits to those in producer membranes, DL uptake with low alteration represents a flow of environmental information through food webs. Finally, some lipid effects may be particularly clear during periods of physical hardship, such as starvation, infection, reproduction, or hibernation. They may even drive life-history decisions as to whether or not to hibernate or to reproduce.

### Retaining structurally important lipids

FA can be stored as triacylglycerides and used as fuel, sterols can be stored as sterol esters but the degree to which they can be used for energy production is not clear. Given that specific lipids can limit fitness (Table 1), it can be beneficial to shield them from being fed into energy metabolism, even during times of environmental hardship. Examples include hibernating mammals that retain in storage unsaturated 18:2 FA that help to withstand cold temperatures, but use 18:1 or 18:0 FA for metabolism instead (Geiser, 2021), or Tree Swallow chicks that during dietary restriction retain n-3 PUFA in the brain and muscle and avoid metabolic use (Twining et al., 2016). Organ-specific selectivity in sparing lipids has also been shown in fruit flies. When reared as larvae on either yeast or plant lipids, then swapped to the opposite lipid type as adults, larval lipids are retained in the brain. This represents highly specific retention because in the reproductive tract larval lipids are replaced by adult DL and because the replacement is stronger for plant than yeast lipids (Knittelfelder et al., 2020).

### Lipid-selective foraging

Any foraging dedicated to obtaining specific DLs can ultimately evolve only via fitness effects. Natural selection acts primarily on reproductive success, and there are several examples of strong DL effects on reproduction. Elegant recent work on the seven-spot ladybird beetle, *Coccinella septempunctata,* showed that beetles forced to consume an all-prey diet produce few sperm and no offspring (Ugine et al., 2022a). Supplying the beetle with plant sterols or cholesterol rescues sperm count and fertility (Table 1). In the absence of plant sterols, beetles have a heightened preference for plant foods to obtain the sterols required for male reproduction – strong evidence for the idea that DL-related fitness effects drive adaptive foraging. This sterol-dependent appetite is widespread across ladybird species (Ugine et al., 2022b) but it remains to be seen whether fertility costs of sterol depletion drive it in all cases.

Male parasitic wasps *Nasonia vitripennis* whose diet is enriched with linoleic acid – their sex pheromone precursor – attract more females and produce more sperm than unsupplemented control males. These male fitness benefits likely favoured a specific kind of lipid choice by mothers: at oviposition, female *Nasonia* prefer to lay eggs in hosts that are enriched in linoleic acid (Blaul and Ruther, 2011).

### Context-dependent foraging for specific lipids

Given that lipid function depends on environmental conditions, selection is predicted to act on environment-dependent lipid selectivity such that different lipids are preferred under different conditions. For example, worker bees that advertise pollen sources to other bees do so more strongly for pollen sources that contain the PUFA type that is in short supply in the colony (Zarchin et al., 2017). A most impressive example shows that *D. melanogaster* survive freezing temperatures when plant-fed, and flies away after being defrosted from an ice cube (Brankatschk et al., 2018) (https://youtu.be/DVZPsmKEmGk), but under the same temperature die when yeast-fed, likely because they lack PUFA and plant sterols (Carvalho et al., 2010). Choice experiments and sterol analyses show that *D. melanogaster* indeed switch food preferences at low temperature from yeast to plant in both the laboratory (Brankatschk et al., 2018) and in the wild (Knittelfelder et al., 2020). Thus, strong survival differences based on DLs are coupled with adaptive food choice. Other animals, such as crayfish, salmon, rodents, or bear, show changes in their diet-mediated lipid profile before the cold season (Geiser, 2021; Pruitt, 1988; Twining et al., 2021; Vranković et al., 2017), pointing to a possibly analogous and widespread cold-related lipid-selective foraging.

Environment-dependent lipid specificity is not restricted to arthropods that lack the ability to synthesise sterols and PUFA. Many vertebrates cannot synthesise n-3 or n-6 PUFA but benefit from them. Mammals entering hibernation experience fewer wake-up episodes and have substantially extended hibernation periods if they had consumed, and stored, larger amounts of PUFA in their fat depots. The energy saved due to fewer wake-ups likely increases fitness (reviewed in Geiser, 2021). Yet, few studies test whether increased tissue PUFAs arise from specific foraging for PUFA-rich food or are simply a by-product a general increase in lipid intake during the process of winter fattening. The clearest example comes from the Djungarian hamster *Phodopus sungorus*: Pre-hibernating individuals preferred a diet rich in unsaturated FAs at low, but SFAs at high temperatures (Hiebert et al., 2003).

Finally, aquatic insects generally contain more unsaturated FAs than terrestrial insects (Twining et al., 2021). Consequently, aquatic insects are preferred by two bat species before their summer torpor (Schalk and Brigham, 1995) and by insectivorous birds whose chicks growth had been shown to improve on the diet rich in unsaturated FAs (Twining et al., 2019a; Twining et al., 2019b). Interestingly, the improved body mass, immunocompetence, and basal metabolic rate on a high-level n-3 PUFA diet is not simply an effect n-3 PUFA quantity (Twining et al., 2016). In the field, peak abundance of aquatic, but not terrestrial, insects is the best predictor of fledging success (Twining et al., 2016).

What is less clear are the mechanisms of lipid sensing, retention, or selectivity. Reception of specific lipids has been identified to act before ingestion in invertebrates (Ahn et al., 2017; Bernays et al., 1976; Buehlmann et al., 2012; Tsuneto et al., 2020) and vertebrates (Besnard et al., 2016; Liu et al., 2017; Yasumatsu et al., 2019) but post-ingestion perception has also been shown (Behmer et al., 1999). Interestingly post ingestion, both the regulation of feeding (Behmer et al., 1999) but also the more fitness-related development itself (Behmer and Elias, 2000) was determined by the amount of unsuitable phytosterols, not suitable ones.

Together, and regardless of the precise mechanism, this evidence suggests that fitness-related benefits of specific DLs correspond to selectivity for these DLs. Provided at least some genetic basis for the lipid preference, and variation between individuals, natural selection for lipid selectivity is unavoidable. Our prediction is that many species of the examples presented in Table 1 will turn out to show lipid selectivity.

## Ideas and speculation I: Ecological and evolutionary consequences of DL selectivity

**Figure 2.**
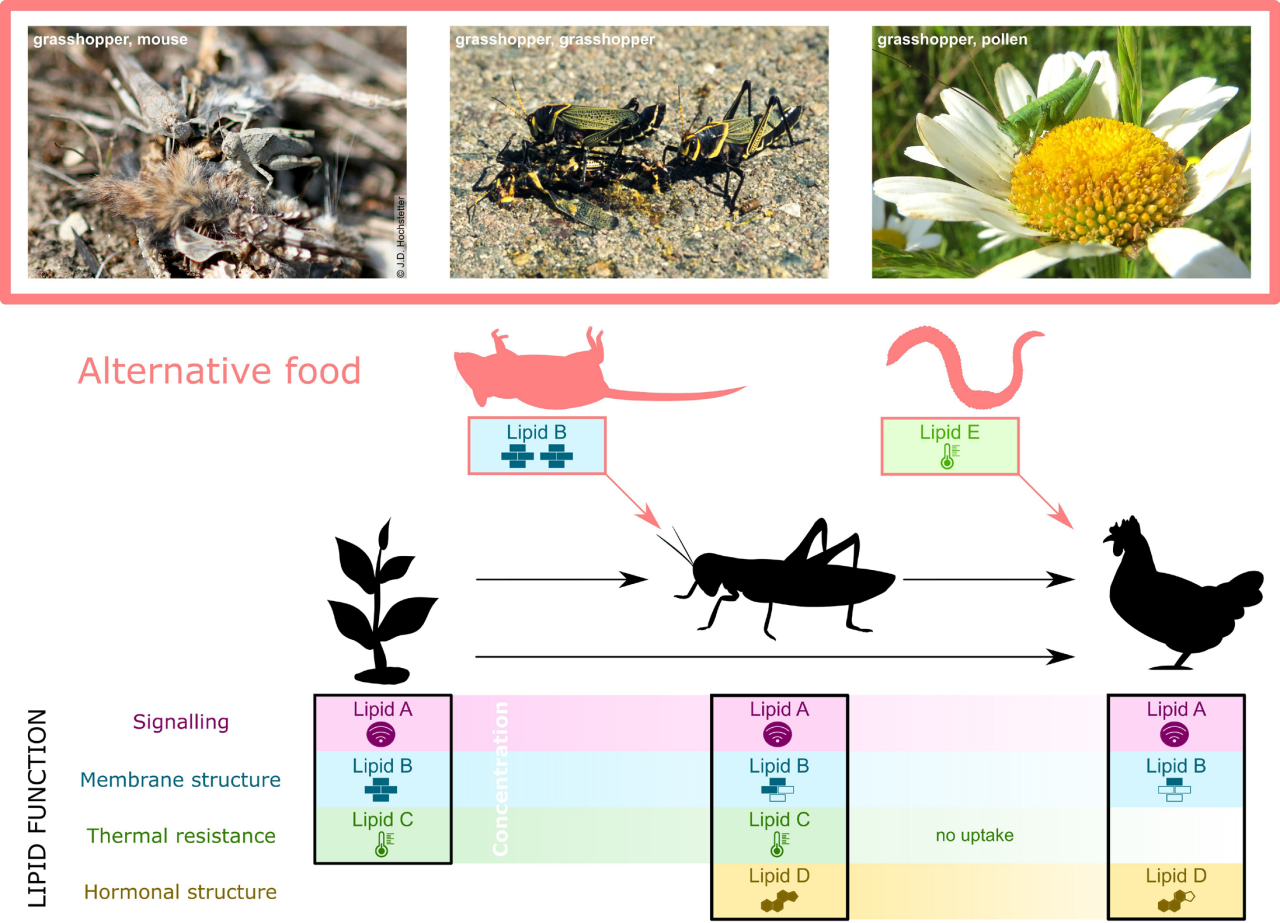
Specific lipid requirements may alter lipid flux through food webs. Producer dietary lipids (DLs) (e.g., plant lipids A to C, left panel, lower section) are the source that consumers use in tailoring their lipid profiles to meet lipid-mediated body functions (black solid lines and icons) other than mere energy provision. For instance, a herbivorous invertebrate acquires three types of DL from plants (Lipids A to C) and produces one itself (Lipid D, middle panel, lower section). The lipid identity may change if, as in some herbivorous insects, dietary plant sterols are converted to cholesterol (reviewed in Behmer and Nes, 2003; Jing and Behmer, 2020). A consumer that feeds on the herbivore and/or the plant acquires three lipids (**A, B, and D**) but is either unable to take up lipid C, or lipid C is available in insufficient concentration (right panel, lower section). Quantitative DL availability changes throughout food webs; in this example, the concentration of all lipids (x-axis, lower section) decreases up the food chain. Qualitative DL availability also changes because Lipids C, D, and E are only found in some species. Variation in quantitative and qualitative DL demand is driven by ecological changes – any resulting deficiencies must be countered by alternative sources of DLs (red arrows and icons). In this example, the low concentration of lipid A in the consumer suffices to enable its role in signalling and no additional uptake is needed (Lavrynenko et al., 2015). Lipid D is a hormone precursor in the consumer but requires structural change to function as a hormone. Under changing environment, such as temperatures, Lipid E, substitutes for the lack of Lipid C due to absorption problems, is needed by the consumer to enable thermal resistance (qualitative limitation). If the quantity of Lipid B is insufficient in the herbivore under an ecological change, an alternative source for Lipid B must be found (quantitative limitation). In nature, many example of such changes in food webs exist, such as scavenging on animal corpses or feeding on pollen by otherwise herbivorous insects (see photographs top section). Ecological Lipidology predicts that acquiring the same lipid from different sources (Lipid B from mouse carcass) or different but functionally similar lipids (Lipid E replacing Lipid C in the chicken) will mainly occur when the environment changes.

Environment-dependent lipid selectivity has substantial ecological consequences that range from the alteration of food webs (Figure 2), the evolution of species differences along the specialism-generalism continuum (Figure 2) to sex-specific evolution. It also offers a new perspective on the explanation of species diversity as detailed below.

### Lipid specificity can alter food webs

Carbohydrates, proteins, and even micronutrients can be obtained from most diets (provided sufficient quantities are eaten), specific lipids only from specific sources. The Ecological Lipidology approach as well as existing nutritional frameworks (Raubenheimer et al., 2009) therefore predict that (1) lipids play a particularly important role in driving alternative foraging strategies (Figure 2) (alongside other nutrients; Simpson and Raubenheimer, 2012), and (2) consumers that turn to alternative food sources mainly do so when ecological changes lead to their preferred DL being in short supply (Figure 2). These alternative strategies substantially alter food webs (Figure 2): Predators turn omnivores (Ugine et al., 2022b), and herbivores turn facultative necrophages, cannibals, or coprophages, or engage in predation or blood sucking. Hundreds of anecdotal observations and photographs of such unusual food switches exist. That these may, in fact, not be anecdotal but driven by the desire for specific DLs in at least some cases (Figure 2), represents a potent, testable hypothesis. Where in the food web these cases are most likely to be expected is currently not clear. The important role of plant-based DLs suggests that lipid selectivity may be pronounced in herbivores (Bernays et al., 1976). However, the general lipid paucity on the top of food webs (Wilder et al., 2013; Figure 2) may extend to specific lipids, in which case ‘unusual’ food switches may occur there more commonly. This will be challenging to detect because top predators in food webs are often generalist and defining a food switch ‘unusual’ is more difficult. In this case, the question arises to what degree the lipid profiles of prey, and not just mere prey availability, drive generalism in the first place.

In freshwater systems, some herbivore consumers have no access to PUFA via algae. However, the free-living stage of specific fungi (Chytridiomycota) parasitise these algae and incorporate algal PUFA, a process known as mycoloop (Kagami et al., 2014). A change in food web structure occurs when herbivores now forage selectively for these fungal spores in order to obtain PUFA (Kagami et al., 2014) and so have effectively turned omnivorous. We suspect that more examples of lipid selectivity exist that drive deviations from the classical herbivore-omnivore-predator classification.

### Does lipid specificity promote range expansion?

Lipid specificity is related to the composition of ecological communities, and thereby to ecosystem function, in two ways. First, as species expand their range, or niche, they enter new communities and encounter DLs new to them. If these DLs have negative organismal or fitness effects (e.g., Table 1) or are not included in their selective filters, expanding species are less likely to become established in ecological communities, making lipid selectivity an important pre-requisite for invasion success. For example, humans likely evolved in a dietary environment of a roughly 1:1 ratio of n-3:n-6 PUFAs (Simopoulos, 2011). Current Western diets provide ratios of >10:1, which are reasoned to contribute to the obesity (Simopoulos, 2006). In entirely natural situations, obesity-related infertility and other disadvantages would cause selection for discrimination against dietary n-6 PUFAs. Obviously, this study is impossible to do; informative examples testing evolutionary consequences of dietary mismatches may come from experimental evolution studies or from studying the response to entirely novel fats, such as artificial trans fats, to which humans also respond suboptimally. A novel food niche is also generated by one of humankind’s breakthroughs, the mastering of fire. Our habit of cooking food inevitably exposes us to an array of DLs that our physiology has not previously been exposed to Nawar, 1984. Research in this area may link Ecological Lipidology to human nutrition research.

Second, lipid selectivity may drive the initial range expansions via the differential effects of DLs on thermal sensitivity. For example, if *Drosophila suzukii* could select lipids by foraging in a similar way to *D. melanogaster* (Knittelfelder et al., 2020) and other animals, the spread from tropical regions into temperate Europe might be augmented by an increased uptake of plant lipids. Similar arguments apply to the above-mentioned examples of hibernating mammals. These may inhabit northern regions only because they are able to select lipids that facilitate hibernation and cold resistance.

### Sex differences in lipid specificity

Males and females of consumers differ in lipid body content and lipid use (Gilbert and Schneiderman, 1961; Scheitz et al., 2013). In many species, females have more storage lipids, higher metabolic lipid turnover, and higher recruitment from storage (Scheitz et al., 2013). In *Daphnia*, both sexes show similar growth rates with increased dietary cholesterol or PUFA (C20:5, EPA) supply but adding EPA plus cholesterol generates stronger somatic growth in females than males (Martin-Creuzburg et al., 2018). Lipid profiles also differ between the sexes. *D. melanogaster* males have more unsaturated FA and a higher phosphatidylcholine/phosphatidyl-ethanolamine ratio (Scheitz et al., 2013). Sexual differences in a process as conserved as lipid use are interesting because they suggest differences in the genetics of lipid processing (Scheitz et al., 2013). Evolutionary change can be very rapid if the trait optima do not coincide for both sexes (intersexual intragenomic conflict). For example, the large demand of sterols for egg development predicts that traits evolve in females that facilitate foraging for, or more efficient use of, different sterols but less in males (unless sterols affect fertility; Ugine et al., 2022b). Consistent with this idea, *D. melanogaster* females, whose DLs were switched from yeast to plant, incorporate large amounts of yeast-derived ergosterol into their reproductive tract whereas males do not (Knittelfelder et al., 2020). Sex differences in lipid processing have obvious important implications not only for human nutrition research, but also for evolutionary biology because diversifying selection is often assigned to males whereas in lipid processing females seem to diversify (Scheitz et al., 2013).

### Macroevolution – sterol auxotrophy as an evolutionary innovation driving diversification?

The last common eukaryotic ancestor was likely able to synthesise sterols (Desmond and Gribaldo, 2009) and most extant Eukaryota and some Prokaryota have retained sterol synthesis (Desmond and Gribaldo, 2009). However, insects and nematodes have become sterol auxotrophs. Sterol auxotrophy may, therefore, be related to a most pertinent question in biology – why are insects (Mayhew, 2018) and possibly nematodes (Smythe et al., 2019) mega-diverse? Species diversity is driven by evolutionary innovations that reduce extinction more than by those that increase speciation; it increases during niche expansions, including the utilisation of novel resources (Mayhew, 2018). We propose sterol auxotrophy as a novel key innovation that increased niche expansion and reduced extinction. The growth and fitness benefits of switching off the sterol-producing machinery are very large: biotechnological yeast production (i.e., offspring number, or fitness) increases manifold even when only parts of sterol production pathways are switched off (e.g., Johnston et al., 2020). If using dietary sterols allowed abandoning the sterol production in nature and resources for defence and reproduction were freed, extinction risks may have been generally reduced but especially so in changing temperatures. The large costs of sterol production make it also unlikely that multiple sterol production pathways have evolved that equipped membranes with different sterols to resist different temperatures. Sterol auxotrophy likely was the much cheaper option whereby sterols were taken up in response to temperature (Brankatschk et al., 2018; Knittelfelder et al., 2020). Sterol independence would, in turn, have allowed insects and nematodes to colonise low-oxygen but sterol-rich environments, such as soil and rotting organic material. Sterol auxotrophy will, of course, not be the only process associated with species diversity and our hypothesis requires further refinements. For example, while arthropods and nematodes form the clade Ecdysozoa, sterol auxotrophy may have evolved independently in both groups. This idea would be consistent with the retention of the full set of cholesterol-producing genes (Zhang et al., 2019) in species-poor related sister clades, such as the tardigrades or, depending on the phylogenetic hypothesis (Dunn et al., 2014), the Scalidophora. On the other hand, there are some non-ecdysozoan taxa that have lost cholesterol-producing genes (Zhang et al., 2019) but their ability to produce other sterols is unknown.

## Ideas and speculation II: A biomedical relevance for Ecological Lipidology?

Much of current lipid cell biology research is based on few cell lines derived from the muscle tissue of only a few individuals (Pradas et al., 2018), and most experiments are carried out at 37°C, the core body temperature of most mammals. Given that lipid profiles can influence thermal responses, lipid research might benefit from examining the lipid profile of tissues that are not at 37°C. For example, the legs of birds (Steen and Steen, 2018) or toes and digits of humans are up to 10°C cooler than the body core temperature for long periods of time. Differences in the lipid profile between these tissues and those at core temperature are currently unknown. The sperm of birds and mammals largely and consistently also differ in FA composition (Surai et al., 2007) and temperature. In the external scrotum of mammals, sperm are up to 8° degrees below body temperature (~30°C) whereas in birds sperm are at body temperature (~40°C). Reproductive biology and medicine may benefit from such insights as well as organ preservation strategies in transplantations. This ecological view of the lipidome may also contribute to the unresolved question why each cell type possesses its own lipid profile and integrates DLs at different rates.

Research in neurobiology and behaviour will also benefit from an ecological view on lipids. DLs affect the neuronal lipid profile in insects (Carvalho et al., 2012), and are essential for the normal development and function of the central nervous system in vertebrates (Cole et al., 2010; González et al., 2010). New evidence shows that the saturation of FAs determines neuronal function in *D. melanogaster*: Experimentally decreasing the saturation of FA in the neurons leads to what is observed after consuming plant lipids: individuals predictably choose lower temperatures (Suito et al., 2020). As alterations of the brain lipidome are hallmarks of neurodegenerative diseases and neuronal aging (Huynh et al., 2020), DL interventions may have a yet undisclosed therapeutic potential.

Many pathways of lipid processing are molecularly or functionally conserved across the animal kingdom, such as the lipoprotein system, LDL receptors, desaturases, or lipases. Therefore, *Drosophila* (sterol and PUFA auxotroph) and *Caenorhabditis elegans* (sterol auxotroph) represent potent models to assess health impacts, metabolic malfunction, and other organismal consequences of lipid function in cell membranes. In reverse, biomedical research on the genetic causes of diseases that affect lipid metabolism may help to explain selection pressures that shape intraspecific genetic variation for lipid processing enzymes in the wild (Twining et al., 2021) or even lipid selectivity.

### Conclusion

High-throughput, high-precision mass spectrometry of lipids coupled with functional analyses has revealed that origin and quantity of lipids available to organisms have profound impacts on cellular functions. These functions – energy provision, signalling, and membrane structure (Figure 1) – when scaling up to cause health, survival, and fitness differences between organisms (Table 1) translate into food web changes (Figure 2) and range expansions in ecosystems and drive the evolution of foraging selectivity for specific lipids. Ecological Lipidology affects our view on sex differences and lifestyle impacts on human nutrition, on how organisms respond to environmental change and provides a hypothesis to explain species diversification. Biomedical research should harness the predictive power of Ecological Lipidology to develop DL-based therapies for a range of diseases.

## References

[bib1] Ahn JE, Chen Y, Amrein H (2017). Molecular basis of fatty acid taste in *Drosophila*. eLife.

[bib2] Awadin WF, Eladl AH, El-Shafei RA, El-Adl MA, Aziza AE, Ali HS, Saif MA (2020). Effect of omega-3 rich diet on the response of japanese quails (coturnix coturnix japonica) infected with newcastle disease virus or avian influenza virus H9N2. Comparative Biochemistry and Physiology. Toxicology & Pharmacology.

[bib3] Bajracharya R, Ballard JWO (2018). Dietary management and physical exercise can improve climbing defects and mitochondrial activity in *Drosophila melanogaster* parkin null mutants. Fly.

[bib4] Behmer ST, Elias DO, Bernays EA (1999). Post-ingestive feedbacks and associative learning regulate the intake of unsuitable sterols in a generalist grasshopper. The Journal of Experimental Biology.

[bib5] Behmer ST, Elias DO (2000). Sterol metabolic constraints as a factor contributing to the maintenance of diet mixing in grasshoppers (orthoptera: acrididae). Physiological and Biochemical Zoology.

[bib6] Behmer ST, Nes WD (2003). Insect sterol nutrition and physiology: a global overview. Adv. Insect Physiol.

[bib7] Bernays EA, Blaney WM, Chapman RF, Cook AG, Bernays EA (1976). In The Host-Plant in Relation to Insect Behaviour and Reproduction.

[bib8] Besnard P, Passilly-Degrace P, Khan NA (2016). Taste of fat: A sixth taste modality?. Physiological Reviews.

[bib9] Blaul B, Ruther J (2011). How parasitoid females produce sexy sons: A causal link between oviposition preference, dietary lipids and mate choice in nasonia. Proceedings. Biological Sciences.

[bib10] Blesbois E, Lessire M, Grasseau I, Hallouis JM, Hermier D (1997). Effect of dietary fat on the fatty acid composition and fertilizing ability of fowl semen. Biology of Reproduction.

[bib11] Brankatschk M, Gutmann T, Knittelfelder O, Palladini A, Prince E, Grzybek M, Brankatschk B, Shevchenko A, Coskun Ü, Eaton S (2018). A temperature-dependent switch in feeding preference improves *Drosophila* development and survival in the cold. Developmental Cell.

[bib12] Buehlmann C, Hansson BS, Knaden M (2012). Path integration controls nest-plume following in desert ants. Current Biology.

[bib13] Caron B, Mark AE, Poger D (2014). Some like it hot: the effect of sterols and hopanoids on lipid ordering at high temperature. The Journal of Physical Chemistry Letters.

[bib14] Carvalho M, Schwudke D, Sampaio JL, Palm W, Riezman I, Dey G, Gupta GD, Mayor S, Riezman H, Shevchenko A, Kurzchalia TV, Eaton S (2010). Survival strategies of a sterol auxotroph. Development.

[bib15] Carvalho M, Sampaio JL, Palm W, Brankatschk M, Eaton S, Shevchenko A (2012). Effects of diet and development on the *Drosophila* lipidome. Molecular Systems Biology.

[bib16] Chen Y, Hagopian K, McDonald RB, Bibus D, López-Lluch G, Villalba JM, Navas P, Ramsey JJ (2012). The influence of dietary lipid composition on skeletal muscle mitochondria from mice following 1 month of calorie restriction. The Journals of Gerontology.

[bib17] Choi LS, Shi C, Ashraf J, Sohrabi S, Murphy CT (2021). Oleic acid protects *caenorhabditis* mothers from mating-induced death and the cost of reproduction. Frontiers in Cell and Developmental Biology.

[bib18] Clayton RB (1964). THE utilization of sterols by insects. Journal of Lipid Research.

[bib19] Cole GM, Ma QL, Frautschy SA (2010). Dietary fatty acids and the aging brain. Nutrition Reviews.

[bib20] Coskun Ü, Simons K (2012). Cell membranes: the lipid perspective. Structure.

[bib21] Daimiel-Ruiz L, Klett-Mingo M, Konstantinidou V, Micó V, Aranda JF, García B, Martínez-Botas J, Dávalos A, Fernández-Hernando C, Ordovás JM (2015). Dietary lipids modulate the expression of mir-107, a mirna that regulates the circadian system. Molecular Nutrition & Food Research.

[bib22] de Carvalho C, Caramujo MJ (2018). The various roles of fatty acids. Molecules.

[bib23] DeCoffe D, Quin C, Gill SK, Tasnim N, Brown K, Godovannyi A, Dai C, Abulizi N, Chan YK, Ghosh S, Gibson DL (2016). Dietary lipid type, rather than total number of calories, alters outcomes of enteric infection in mice. The Journal of Infectious Diseases.

[bib24] Desmond E, Gribaldo S (2009). Phylogenomics of sterol synthesis: insights into the origin, evolution, and diversity of a key eukaryotic feature. Genome Biology and Evolution.

[bib25] Dunn CW, Giribet G, Edgecombe GD, Hejnol A (2014). Animal phylogeny and its evolutionary implications. Annual Review of Ecology, Evolution, and Systematics.

[bib26] Eigenbrode SD, Espelie KE (1995). Effects of plant epicuticular lipids on insect herbivores. Annual Review of Entomology.

[bib27] Ejsing CS, Sampaio JL, Surendranath V, Duchoslav E, Ekroos K, Klemm RW, Simons K, Shevchenko A (2009). Global analysis of the yeast lipidome by quantitative shotgun mass spectrometry. PNAS.

[bib28] Fuller HL, Rendon M (1977). Energetic efficiency of different dietary fats for growth of young chicks. Poultry Science.

[bib29] Garda HA, Bernasconi AM, Brenner RR, Aguilar F, Soto MA, Sotomayor CP (1997). Effect of polyunsaturated fatty acid deficiency on dipole relaxation in the membrane interface of rat liver microsomes. Biochimica et Biophysica Acta.

[bib30] Geiser F (1990). Influence of polyunsaturated and saturated dietary lipids on adipose tissue, brain and mitochondrial membrane fatty acid composition of a mammalian hibernator. Biochimica et Biophysica Acta.

[bib31] Geiser F, Geiser F (2021). Ecological Physiology of Daily Torpor and Hibernation.

[bib32] Gilbert LI, Schneiderman HA (1961). The content of juvenile hormone and lipid in lepidoptera: sexual differences and developmental changes. General and Comparative Endocrinology.

[bib33] Giraud MN, Motta C, Boucher D, Grizard G (2000). Membrane fluidity predicts the outcome of cryopreservation of human spermatozoa. Human Reproduction.

[bib34] González S, Huerta JM, Fernández S, Patterson AM, Lasheras C (2010). The relationship between dietary lipids and cognitive performance in an elderly population. International Journal of Food Sciences and Nutrition.

[bib35] Guo R, Reinhardt K (2020). Dietary polyunsaturated fatty acids affect volume and metabolism of *Drosophila melanogaster* sperm. Journal of Evolutionary Biology.

[bib36] Harayama T, Riezman H (2018). Understanding the diversity of membrane lipid composition. Nature Reviews. Molecular Cell Biology.

[bib37] Harris WS, Tintle NL, Imamura F, Qian F, Korat AVA, Marklund M, Djoussé L, Bassett JK, Carmichael PH, Chen YY, Hirakawa Y, Küpers LK, Laguzzi F, Lankinen M, Murphy RA, Samieri C, Senn MK, Shi P, Virtanen JK, Brouwer IA, Chien KL, Eiriksdottir G, Forouhi NG, Geleijnse JM, Giles GG, Gudnason V, Helmer C, Hodge A, Jackson R, Khaw KT, Laakso M, Lai H, Laurin D, Leander K, Lindsay J, Micha R, Mursu J, Ninomiya T, Post W, Psaty BM, Risérus U, Robinson JG, Shadyab AH, Snetselaar L, Sala-Vila A, Sun Y, Steffen LM, Tsai MY, Wareham NJ, Wood AC, Wu JHY, Hu F, Sun Q, Siscovick DS, Lemaitre RN, Mozaffarian D, Fatty Acids and Outcomes Research Consortium FORCE (2021). Blood n-3 fatty acid levels and total and cause-specific mortality from 17 prospective studies. Nature Communications.

[bib38] Hazel JR (1995). Thermal adaptation in biological membranes: is homeoviscous adaptation the explanation?. Annual Review of Physiology.

[bib39] Hiebert SM, Hauser K, Ebrahim AJ (2003). Djungarian hamsters exhibit temperature-dependent dietary fat choice in long days. Physiological and Biochemical Zoology.

[bib40] Hill VL, Florant GL (2000). The effect of a linseed oil diet on hibernation in yellow-bellied marmots (marmota flaviventris). Physiology & Behavior.

[bib41] Hryhorczuk C, Florea M, Rodaros D, Poirier I, Daneault C, Des Rosiers C, Arvanitogiannis A, Alquier T, Fulton S (2016). Dampened mesolimbic dopamine function and signaling by saturated but not monounsaturated dietary lipids. Neuropsychopharmacology: Official Publication of the American College of Neuropsychopharmacology.

[bib42] Hulbert AJ (2005). On the importance of fatty acid composition of membranes for aging. Journal of Theoretical Biology.

[bib43] Hulbert AJ, Abbott SK (2011). Nutritional ecology of essential fatty acids: an evolutionary perspective. Australian Journal of Zoology.

[bib44] Hulbert AJ, Kelly MA, Abbott SK (2014). Polyunsaturated fats, membrane lipids and animal longevity. Journal of Comparative Physiology. B, Biochemical, Systemic, and Environmental Physiology.

[bib45] Huynh K, Lim WLF, Giles C, Jayawardana KS, Salim A, Mellett NA, Smith AAT, Olshansky G, Drew BG, Chatterjee P, Martins I, Laws SM, Bush AI, Rowe CC, Villemagne VL, Ames D, Masters CL, Arnold M, Nho K, Saykin AJ, Baillie R, Han X, Kaddurah-Daouk R, Martins RN, Meikle PJ (2020). Concordant peripheral lipidome signatures in two large clinical studies of alzheimer’s disease. Nature Communications.

[bib46] Ilić M, Werner C, Fink P (2019). Equal relevance of omega‐3 and omega‐6 polyunsaturated fatty acids for the fitness of daphnia spp. Limnology and Oceanography.

[bib47] Jie F, Yang X, Wu L, Wang M, Lu B (2019). Linking phytosterols and oxyphytosterols from food to brain health: origins, effects, and underlying mechanisms. Critical Reviews in Food Science and Nutrition.

[bib48] Jing X, Behmer ST (2020). Insect sterol nutrition: physiological mechanisms, ecology, and applications. Annual Review of Entomology.

[bib49] Johnston EJ, Moses T, Rosser SJ (2020). The wide‐ranging phenotypes of ergosterol biosynthesis mutants, and implications for microbial cell factories. Yeast.

[bib50] Kagami M, Miki T, Takimoto G (2014). Mycoloop: chytrids in aquatic food webs. Frontiers in Microbiology.

[bib51] Kainz M, Brett MT, Arts MT (2009). Lipids in Aquatic Ecosystems.

[bib52] Kaliszewicz A, Jarząbek K, Szymańska J, Karaban K, Sierakowski M (2018). Alpha-linolenic acid, but not palmitic acid, negatively impacts survival, asexual reproductive rate, and clonal offspring size in hydra oligactis. Lipids.

[bib53] Knittelfelder O, Prince E, Sales S, Fritzsche E, Wöhner T, Brankatschk M, Shevchenko A (2020). Sterols as dietary markers for *Drosophila melanogaster*. Biochimica et Biophysica Acta. Molecular and Cell Biology of Lipids.

[bib54] Kostal V, Hans R (2010). In Low Temperature Biology of Insects.

[bib55] Lavrynenko O, Rodenfels J, Carvalho M, Dye NA, Lafont R, Eaton S, Shevchenko A (2015). The ecdysteroidome of *Drosophila*: influence of diet and development. Development.

[bib56] Levental KR, Malmberg E, Symons JL, Fan YY, Chapkin RS, Ernst R, Levental I (2020). Lipidomic and biophysical homeostasis of mammalian membranes counteracts dietary lipid perturbations to maintain cellular fitness. Nature Communications.

[bib57] Levi L, Wang Z, Doud MK, Hazen SL, Noy N (2015). Saturated fatty acids regulate retinoic acid signalling and suppress tumorigenesis by targeting fatty acid-binding protein 5. Nature Communications.

[bib58] Lien EC, Westermark AM, Zhang Y, Yuan C, Li Z, Lau AN, Sapp KM, Wolpin BM, Vander Heiden MG (2021). Low glycaemic diets alter lipid metabolism to influence tumour growth. Nature.

[bib59] Lin X, Gorfe AA, Levental I (2018). Protein partitioning into ordered membrane domains: insights from simulations. Biophysical Journal.

[bib60] Lionetti L, Mollica MP, Donizzetti I, Gifuni G, Sica R, Pignalosa A, Cavaliere G, Gaita M, De Filippo C, Zorzano A, Putti R (2014). High-lard and high-fish-oil diets differ in their effects on function and dynamic behaviour of rat hepatic mitochondria. PLOS ONE.

[bib61] Liu H, Xu Y, Wang Y, Zhong S, Wang M, Lin P, Li H, Liu Z (2017). Cd36 is a candidate lipid sensor involved in the sensory detection of fatty acid in zebrafish. Physiology & Behavior.

[bib62] Liu JF, Xia JJ, Nie KL, Wang F, Deng L (2019). Outline of the biosynthesis and regulation of ergosterol in yeast. World Journal of Microbiology & Biotechnology.

[bib63] López-Fandiño R (2020). Role of dietary lipids in food allergy. Critical Reviews in Food Science and Nutrition.

[bib64] Ludwig DS, Willett WC, Volek JS, Neuhouser ML (2018). Dietary fat: from foe to friend?. Science.

[bib65] Martin-Creuzburg D, Massier T, Wacker A (2018). Sex-specific differences in essential lipid requirements of daphnia magna. Frontiers in Ecology and Evolution.

[bib66] Mayhew PJ (2018). Explaining global insect species richness: lessons from a decade of macroevolutionary entomology. Entomologia Experimentalis et Applicata.

[bib67] Meln I, Wolff G, Gajek T, Koddebusch J, Lerch S, Harbrecht L, Hong W, Bayindir-Buchhalter I, Krunic D, Augustin HG, Vegiopoulos A (2019). Dietary calories and lipids synergistically shape adipose tissue cellularity during postnatal growth. Molecular Metabolism.

[bib68] Moussavi N, Gavino V, Receveur O (2008). Could the quality of dietary fat, and not just its quantity, be related to risk of obesity?. Obesity.

[bib69] Naudí A, Jové M, Ayala V, Portero-Otín M, Barja G, Pamplona R (2013). Membrane lipid unsaturation as physiological adaptation to animal longevity. Frontiers in Physiology.

[bib70] Nawar WW (1984). Chemical changes in lipids produced by thermal processing. Journal of Chemical Education.

[bib71] Obniski R, Sieber M, Spradling AC (2018). Dietary lipids modulate notch signaling and influence adult intestinal development and metabolism in *Drosophila*. Developmental Cell.

[bib72] Oh DY, Talukdar S, Bae EJ, Imamura T, Morinaga H, Fan W, Li P, Lu WJ, Watkins SM, Olefsky JM (2013). GPR120 is an omega-3 fatty acid receptor mediating potent anti-inflammatory and insulin-sensitizing effects. Cell.

[bib73] O’Rourke EJ, Kuballa P, Xavier R, Ruvkun G (2013). ω-6 polyunsaturated fatty acids extend life span through the activation of autophagy. Genes & Development.

[bib74] Perez MA, Magtanong L, Dixon SJ, Watts JL (2013). Dietary lipids induce ferroptosis in *Caenorhabditis elegans* and human cancer cells. Developmental Cell.

[bib75] Pradas I, Huynh K, Cabré R, Ayala V, Meikle PJ, Jové M, Pamplona R (2018). Lipidomics reveals a tissue-specific fingerprint. Frontiers in Physiology.

[bib76] Pruitt NL (1988). Membrane lipid composition and overwintering strategy in thermally acclimated crayfish. American Journal of Physiology-Regulatory, Integrative and Comparative Physiology.

[bib77] Rahman MM, Gasparini C, Turchini GM, Evans JP (2014). Experimental reduction in dietary omega-3 polyunsaturated fatty acids depresses sperm competitiveness. Biology Letters.

[bib78] Raubenheimer D, Simpson SJ, Mayntz D (2009). Nutrition, ecology and nutritional ecology: toward an integrated framework. Functional Ecology.

[bib79] Rogowska A, Szakiel A (1988). The role of sterols in plant response to abiotic stress. Phytochemistry Reviews.

[bib80] Ruiz T, Koussoroplis A, Danger M, Aguer J, Morel‐Desrosiers N, Bec A, Auer S (1988). Quantifying the energetic cost of food quality constraints on resting metabolism to integrate nutritional and metabolic ecology. Ecology Letters.

[bib81] Saez F, Drevet JR (2019). Dietary cholesterol and lipid overload: impact on male fertility. Oxidative Medicine and Cellular Longevity.

[bib82] Schalk G, Brigham RM (1995). Prey selection by insectivorous bats: are essential fatty acids important?. Canadian Journal of Zoology.

[bib83] Scheitz CJF, Guo Y, Early AM, Harshman LG, Clark AG (2013). Heritability and inter-population differences in lipid profiles of *Drosophila melanogaster*. PLOS ONE.

[bib84] Schoeler M, Caesar R (2019). Dietary lipids, gut microbiota and lipid metabolism. Reviews in Endocrine & Metabolic Disorders.

[bib85] Schultzhaus JN, Carney GE (2017). Dietary protein content alters both male and female contributions to *Drosophila melanogaster* female post-mating response traits. Journal of Insect Physiology.

[bib86] Schultzhaus JN, Bennett CJ, Iftikhar H, Yew JY, Mallett J, Carney GE (2018). High fat diet alters *Drosophila melanogaster* sexual behavior and traits: decreased attractiveness and changes in pheromone profiles. Scientific Reports.

[bib87] Senyilmaz D, Virtue S, Xu X, Tan CY, Griffin JL, Miller AK, Vidal-Puig A, Teleman AA (2015). Regulation of mitochondrial morphology and function by stearoylation of TFR1. Nature.

[bib88] Senyilmaz Tiebe D, Pfaff DH, Virtue S, Schwarz KV, Fleming T, Altamura S, Muckenthaler MU, Okun JG, Vidal Puig A, Nawroth P, Teleman AA (2018). Dietary stearic acid regulates mitochondria in vivo in humans. Nature Communications.

[bib89] Simandle ET, Espinoza RE, Nussear KE, Tracy CR (2001). Lizards, lipids, and dietary links to animal function. Physiological and Biochemical Zoology.

[bib90] Simopoulos AP (2006). Evolutionary aspects of diet, the omega-6/omega-3 ratio and genetic variation: nutritional implications for chronic diseases. Biomedicine & Pharmacotherapy = Biomedecine & Pharmacotherapie.

[bib91] Simopoulos AP (2011). Evolutionary aspects of diet: the omega-6/omega-3 ratio and the brain. Molecular Neurobiology.

[bib92] Simpson SJ, Raubenheimer D (2012). The Nature of Nutrition: A Unifying Framework from Animal Adaptation to Human Obesity.

[bib93] Sinensky M (1974). Homeoviscous adaptation--a homeostatic process that regulates the viscosity of membrane lipids in *Escherichia coli*. PNAS.

[bib94] Smythe AB, Holovachov O, Kocot KM (2019). Improved phylogenomic sampling of free-living nematodes enhances resolution of higher-level nematode phylogeny. BMC Evolutionary Biology.

[bib95] Stachowiak JC, Brodsky FM, Miller EA (2013). A cost-benefit analysis of the physical mechanisms of membrane curvature. Nature Cell Biology.

[bib96] Steck TL, Lange Y (2018). Transverse distribution of plasma membrane bilayer cholesterol: picking sides. Traffic.

[bib97] Steen I, Steen JB (2018). The importance of the legs in the thermoregulation of birds. Acta Physiologica Scandinavica.

[bib98] Stickney RR, Andrews JW (1972). Effects of dietary lipids on growth, food conversion, lipid and fatty acid composition of channel catfish. The Journal of Nutrition.

[bib99] Stinkens R, Goossens GH, Jocken JWE, Blaak EE (2015). Targeting fatty acid metabolism to improve glucose metabolism. Obesity Reviews.

[bib100] Suito T, Nagao K, Takeuchi K, Juni N, Hara Y, Umeda M (2020). Functional expression of δ12 fatty acid desaturase modulates thermoregulatory behaviour in *Drosophila*. Scientific Reports.

[bib101] Sullivan EM, Pennington ER, Sparagna GC, Torres MJ, Neufer PD, Harris M, Washington J, Anderson EJ, Zeczycki TN, Brown DA, Shaikh SR (2018). Docosahexaenoic acid lowers cardiac mitochondrial enzyme activity by replacing linoleic acid in the phospholipidome. The Journal of Biological Chemistry.

[bib102] Surai PF, Fujihara N, Speake BK, Brillard J-P, Wishart GJ, Sparks NHC (2007). Polyunsaturated fatty acids, lipid peroxidation and antioxidant protection in avian semen - review -. Asian-Australasian Journal of Animal Sciences.

[bib103] Surma MA, Gerl MJ, Herzog R, Helppi J, Simons K, Klose C (2021). Mouse lipidomics reveals inherent flexibility of a mammalian lipidome. Scientific Reports.

[bib104] Tagliafico A, Rudd D, Rangel MS, Kelaher BP, Christidis L, Cowden K, Scheffers SR, Benkendorff K (2007). Lipid-enriched diets reduce the impacts of thermal stress in corals. Marine Ecology Progress Series.

[bib105] Tidwell JH, Coyle S, Bright LA (2007). Effects of different types of dietary lipids on growth and fatty acid composition of largemouth bass. North American Journal of Aquaculture.

[bib106] Tognini P, Murakami M, Liu Y, Eckel-Mahan KL, Newman JC, Verdin E, Baldi P, Sassone-Corsi P (2017). Distinct circadian signatures in liver and gut clocks revealed by ketogenic diet. Cell Metabolism.

[bib107] Tsuneto K, Endo H, Shii F, Sasaki K, Nagata S, Sato R (2020). Diet choice: the two-factor host acceptance system of silkworm larvae. PLOS Biology.

[bib108] Turk M, Plemenitaš A, Gunde-Cimerman N (2011). Extremophilic yeasts: plasma-membrane fluidity as determinant of stress tolerance. Fungal Biology.

[bib109] Twining CW, Brenna JT, Lawrence P, Shipley JR, Tollefson TN, Winkler DW (2016). Omega-3 long-chain polyunsaturated fatty acids support aerial insectivore performance more than food quantity. PNAS.

[bib110] Twining CW, Brenna JT, Lawrence P, Winkler DW, Flecker AS, Hairston NG, Allen DC (2019a). Aquatic and terrestrial resources are not nutritionally reciprocal for consumers. Functional Ecology.

[bib111] Twining CW, Shipley JR, Winkler DW, Jeyasingh P (2019b). Aquatic insects rich in omega‐3 fatty acids drive breeding success in a widespread bird. Ecology Letters.

[bib112] Twining CW, Bernhardt JR, Derry AM, Hudson CM, Ishikawa A, Kabeya N, Kainz MJ, Kitano J, Kowarik C, Ladd SN, Leal MC, Scharnweber K, Shipley JR, Matthews B (2021). The evolutionary ecology of fatty-acid variation: implications for consumer adaptation and diversification. Ecology Letters.

[bib113] Ugine TA, Krasnoff SB, Behmer ST (2022a). Omnivory in predatory lady beetles is widespread and driven by an appetite for sterols. Functional Ecology.

[bib114] Ugine TA, Krasnoff SB, Grebenok RJ, Behmer ST, Losey JE, Irwin R (2022b). Prey nutrient content creates omnivores out of predators. Ecology Letters.

[bib115] Vasam G, Reid K, Burelle Y, Menzies KJ, Vasam G (2019). Mitochondria in Obesity and Type 2 Diabetes.

[bib116] Vranković L, Delaš I, Reljić S, Huber Đ, Maltar-Strmečki N, Klobučar K, Krivić G, Stojević Z, Aladrović J (2017). The lipid composition of subcutaneous adipose tissue of brown bears (ursus arctos) in croatia. Physiological and Biochemical Zoology.

[bib117] Wang X, Li W, Li M, Welti R (2006). Profiling lipid changes in plant response to low temperatures. Physiologia Plantarum.

[bib118] Wang B, Rong X, Duerr MA, Hermanson DJ, Hedde PN, Wong JS, Vallim TDA, Cravatt BF, Gratton E, Ford DA, Tontonoz P (2016). Intestinal phospholipid remodeling is required for dietary-lipid uptake and survival on a high-fat diet. Cell Metabolism.

[bib119] Wang DD, Hu FB (2017). Dietary fat and risk of cardiovascular disease: recent controversies and advances. Annual Review of Nutrition.

[bib120] Wathes DC, Abayasekara DRE, Aitken RJ (2017). Polyunsaturated fatty acids in male and female reproduction1. Biology of Reproduction.

[bib121] Weill P, Plissonneau C, Legrand P, Rioux V, Thibault R (2020). May omega-3 fatty acid dietary supplementation help reduce severe complications in covid-19 patients?. Biochimie.

[bib122] Wilder SM, Norris M, Lee RW, Raubenheimer D, Simpson SJ (2013). Arthropod food webs become increasingly lipid-limited at higher trophic levels. Ecology Letters.

[bib123] Yan L, Bai X, Fang Z, Che L, Xu S, Wu D (2013). Effect of different dietary omega-3/omega-6 fatty acid ratios on reproduction in male rats. Lipids in Health and Disease.

[bib124] Yang Q, Vijayakumar A, Kahn BB (2018). Metabolites as regulators of insulin sensitivity and metabolism. Nature Reviews. Molecular Cell Biology.

[bib125] Yasumatsu K, Iwata S, Inoue M, Ninomiya Y (2019). Fatty acid taste quality information via GPR120 in the anterior tongue of mice. Acta Physiologica.

[bib126] Yore MM, Syed I, Moraes-Vieira PM, Zhang T, Herman MA, Homan EA, Patel RT, Lee J, Chen S, Peroni OD, Dhaneshwar AS, Hammarstedt A, Smith U, McGraw TE, Saghatelian A, Kahn BB (2014). Discovery of a class of endogenous mammalian lipids with anti-diabetic and anti-inflammatory effects. Cell.

[bib127] Yu L, Fink BD, Herlein JA, Oltman CL, Lamping KG, Sivitz WI (2014). Dietary fat, fatty acid saturation and mitochondrial bioenergetics. Journal of Bioenergetics and Biomembranes.

[bib128] Zanco B, Mirth CK, Sgrò CM, Piper MDW (2021). A dietary sterol trade-off determines lifespan responses to dietary restriction in *Drosophila melanogaster* females. eLife.

[bib129] Zarchin S, Dag A, Salomon M, Hendriksma HP, Shafir S (2017). Honey bees dance faster for pollen that complements colony essential fatty acid deficiency. Behavioral Ecology and Sociobiology.

[bib130] Zeis B, Buchen I, Wacker A, Martin-Creuzburg D (2019). Temperature-induced changes in body lipid composition affect vulnerability to oxidative stress in daphnia magna. Comparative Biochemistry and Physiology. Part B, Biochemistry & Molecular Biology.

[bib131] Zhang T, Yuan D, Xie J, Lei Y, Li J, Fang G, Tian L, Liu J, Cui Y, Zhang M, Xiao Y, Xu Y, Zhang J, Zhu M, Zhan S, Li S (2019). Evolution of the cholesterol biosynthesis pathway in animals. Molecular Biology and Evolution.

[bib132] Zu P, Koch H, Schwery O, Pironon S, Phillips C, Ondo I, Farrell IW, Nes WD, Moore E, Wright GA, Farman DI, Stevenson PC (2021). Pollen sterols are associated with phylogeny and environment but not with pollinator guilds. The New Phytologist.

